# Sushruta: The Father of Surgery and Ancient Medical Innovations

**DOI:** 10.7759/cureus.70577

**Published:** 2024-09-30

**Authors:** Madhura A Gandhi, Bhagyashri K Patil

**Affiliations:** 1 Central Research Facility, Dr. D. Y. Patil Medical College, Hospital and Research Centre, Dr. D. Y. Patil Vidyapeeth (Deemed to be University), Pune, IND

**Keywords:** anatomy, cadaver dissection, cataract surgery, father of surgery, historical vignette, medicine, plastic and reconstructive surgery, rhinoplasty surgery, sushruta, sushruta samhita

## Abstract

Sushruta, known as the "Father of Surgery," was a pioneering figure in ancient Indian medicine whose contributions have influenced the field of surgery, especially cosmetic surgery and medical practices within the global community. His systematized approach to surgery, with the use of herbal anaesthetics and postoperative care, laid the foundation for surgical practices. Sushruta's emphasis on practical experience, dissection of cadavers, and detailed documentation established standards that continue to underpin surgical education today. The influence of his detailing surgical techniques and surgical instruments extended beyond ancient India, spreading to other parts of the world and shaping the development of reconstructive surgery, orthopaedics, ophthalmology, and many more fields. Sushruta has a rich history of medicine that is imbued with his pioneering spirit, which carried enduring influence. His works have been relevant in fomenting generations of surgeons and medical practitioners into the annals of history and solidifying his place as a timeless figure in the evolution of medical science.

## Introduction and background

Ancient civilizations such as those in India, Greece, Mesopotamia, Egypt, and China have all contributed to the early study of medicine and human anatomy. Among these, the Indian sages, guided by Vedic philosophies and the Ayurvedic tradition, established some of the oldest known medical systems. Indian medicine has predated many other ancient medical traditions by over four centuries. Among these early pioneers, Sushruta is often referred to as the "Father of Surgery" [1], being one of the first pioneers in ancient Indian medicine to contribute to such worthwhile findings that have laid the very foundation of modern-day surgical practices. Flourishing inside the historical town of Kashi around 600 BCE, Sushruta's holistic technique to remedy encompassed an extensive range of fields, consisting of surgery (*Shalya Tantra*), anatomy, beauty and reconstructive surgical treatment, obstetrics and gynaecology, ophthalmology, orthopaedics, traumatology, and pharmacology [2]. Sushruta's teachings were not a newly created aspect within the existing practices of his era; they further went on to lay a foundation that truly influenced the global scenario of medical science. His seminal work, encapsulated in the ancient Indian text "*Sushruta Samhita*" [3], reflects his mastery of surgical techniques and human anatomy, marking him as a pioneering figure in the history of medicine. Sushruta's teachings not only revolutionized medical practices in his time but also influenced modern medicine worldwide.

## Review

Early life and education

Sushruta's early life is enveloped within the wealthy traditions and myths of ancient India. He was born in the sixth century BCE and is recognized as the "Golden Age of Indian Medicine" in the town of Kashi, which stands as one of the oldest constantly inhabited cities. The city has a rich history of medical knowledge dating back to 1500 BCE [2].

Although much about his early life remains shrouded in mystery, it is believed that Sushruta practised and imparted his medical knowledge as a disciple of Dievodasa from the Gurukul of Dhanwantri, who is the revered Lord Deity of Ayurveda [1,4]. Sushruta is closely associated with the lineage of the sage Vishvamitra, one of the greatly venerated seers in the Hindu tradition. In ancient India, medicine was traditionally taught through "Guru-Shishya Parampara," a hereditary model where knowledge was passed orally from the teacher to the pupil [5]. Sushruta's education was deeply rooted in the tradition of Ayurveda (called the science of life), a historic Indian system of medicine that deals with balancing body, mind, and spirit. From a tender age of his life, Sushruta must have been exposed to diverse aspects of medicine, including surgery, pharmacology, and herbal medicine. This period of intense learning and practical exposure inscribed in him a deep sense of the human body and the dynamics involved with surgery. This was when Sushruta started viewing surgery not only as a last resort but as the most important and reforming feature of the field of medicine.

Empowered by the wisdom of his guru, Sushruta took a firm stand and professed surgical practices emphasizing the importance of anatomy and practical surgery through cadaver dissection. This marked a significant shift in his life, as he transitioned from a student of traditional medicine to a revolutionary who would change the very face of surgical procedures.

Challenges in life

Sushruta faced significant cultural and societal challenges, particularly regarding his pioneering practices. During his time, the dissection of human bodies was considered taboo, as it was believed to render practitioners impure, a view strongly criticized by religious and cultural authorities [6]. Despite this, Sushruta remained steadfast in his belief that understanding human anatomy through dissection was crucial for successful surgery. The process of surgery itself was often viewed with suspicion, almost regarded as a last resort rather than a legitimate treatment method.

Sushruta's courage to challenge these norms and his commitment to advancing medical and surgical practices despite substantial opposition highlight his resilience and foresight. His ability to navigate these obstacles was instrumental in establishing his lasting legacy as the father of surgery, transforming medical practices for generations to come.

Foundations of Sushruta's surgical mastery and innovations

Sushruta's contributions to surgery were truly revolutionary. His techniques for cataract surgery, rhinoplasty, and the treatment of fractures and wounds were superior for his time. He pioneered the art of suturing, using materials such as horsehair and plant fibres, and meticulously documented each procedure, instrument, and the necessary postoperative care [5]. His emphasis on precision, safety, and cleanliness set new standards for surgical practices. Although Sushruta made numerous contributions to medicine, the key innovations listed below, detailed in the "*Sushruta Samhita*," highlight his most notable advancements:

Cadaver Dissection and Anatomical Study

Sushruta's advocacy for cadaver dissection was a landmark in his medical practice and education, particularly in a culture that resisted such practices. In the *Sushruta Samhita*, he emphasized the necessity of direct observation through dissection to master human anatomy [6]. Despite Hindu beliefs that regarded the human body as sacred in death, Sushruta navigated these restrictions innovatively. His meticulous method involved submerging bodies in flowing water to aid decomposition and then examining them layer by layer. Sushruta specified the use of only bodies free from disease, not older than 100 years, and not poisoned [7]. After seven nights of decomposition, the body was carefully cleaned and dissected [8]. This approach allowed Sushruta and his students to study anatomical structures in detail, significantly advancing their understanding of human physiology and pathology [9].

After performing dissections with these innovative techniques, Sushruta significantly advanced anatomical research. His observations were not superficial; he classified and described anatomy in comprehensive detail, laying out the relationships between different structures, which enabled a well-rounded understanding of human anatomy [8]. His teaching methods included using clay, wax, and ghee to simulate human tissues, enabling students to practice surgical techniques in a controlled setting before performing them on real patients. This hands-on approach was pioneering for its time and set a new standard for medical education. Emphasizing the importance of anatomy in surgery, he encouraged students to practical experience, detailed anatomical study, and step-by-step surgical instructions, highlighting his influence on future generations of medical practitioners.

Surgical instruments

**Figure 1:**
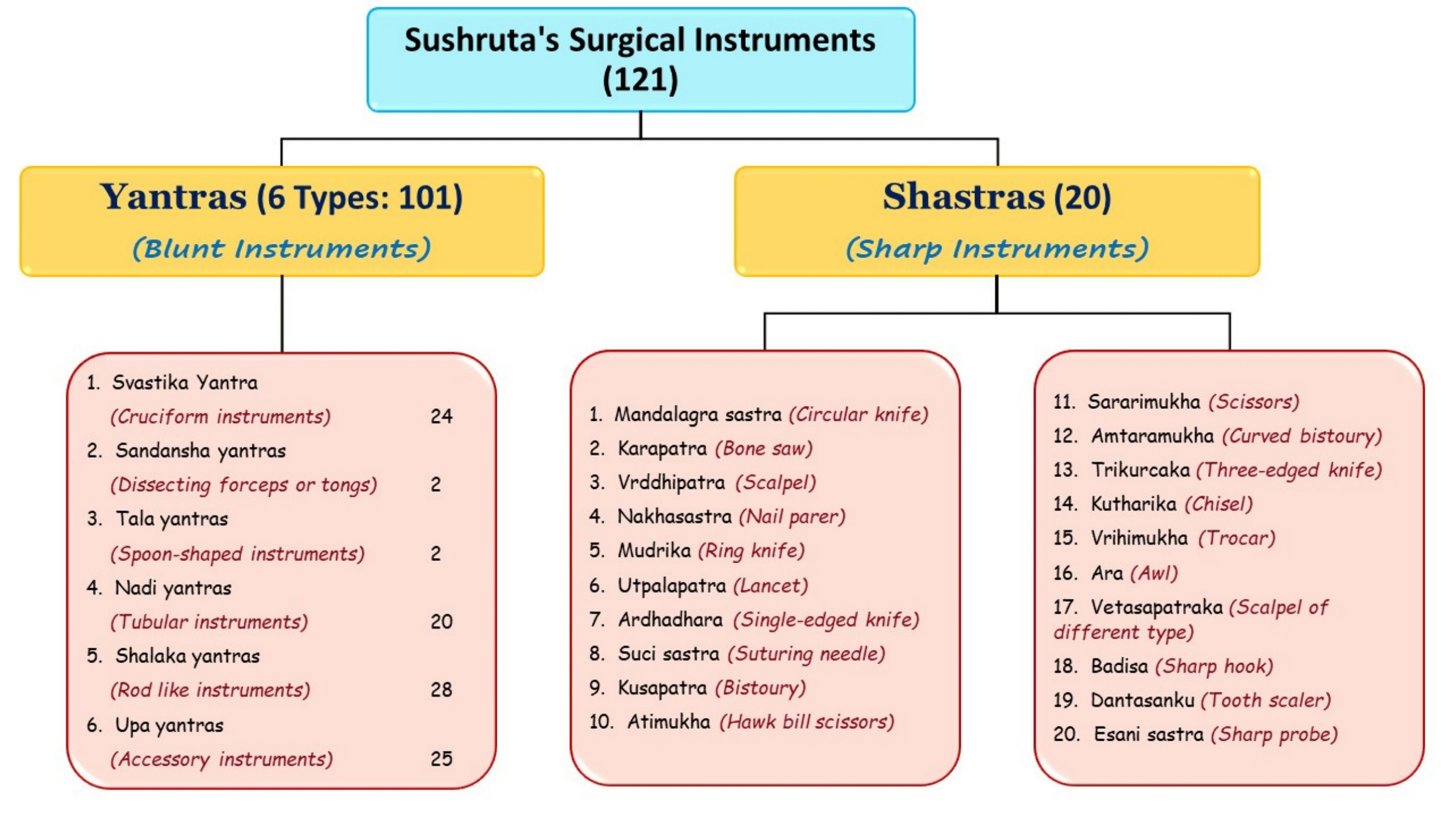
Surgical instruments invented by Sushruta Image source: Image created by the author.

**Figure 2:**
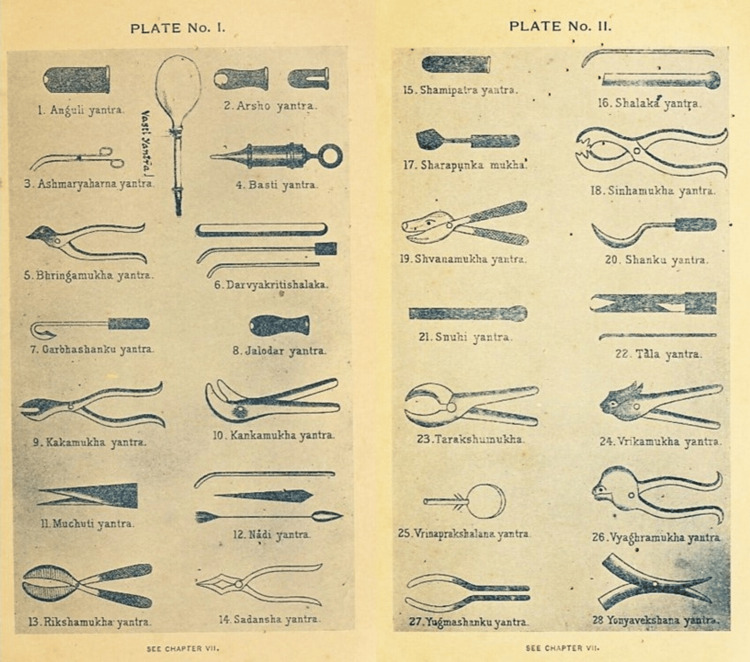
Yantras (Blunt Instruments) invented by Aacharya Sushruta Image source: Screenshots adapted from An English Translation of the Sushruta Samhita, by Kaviraj Kunja Lal Bhishagratna [3], with modifications by the author.

**Figure 3:**
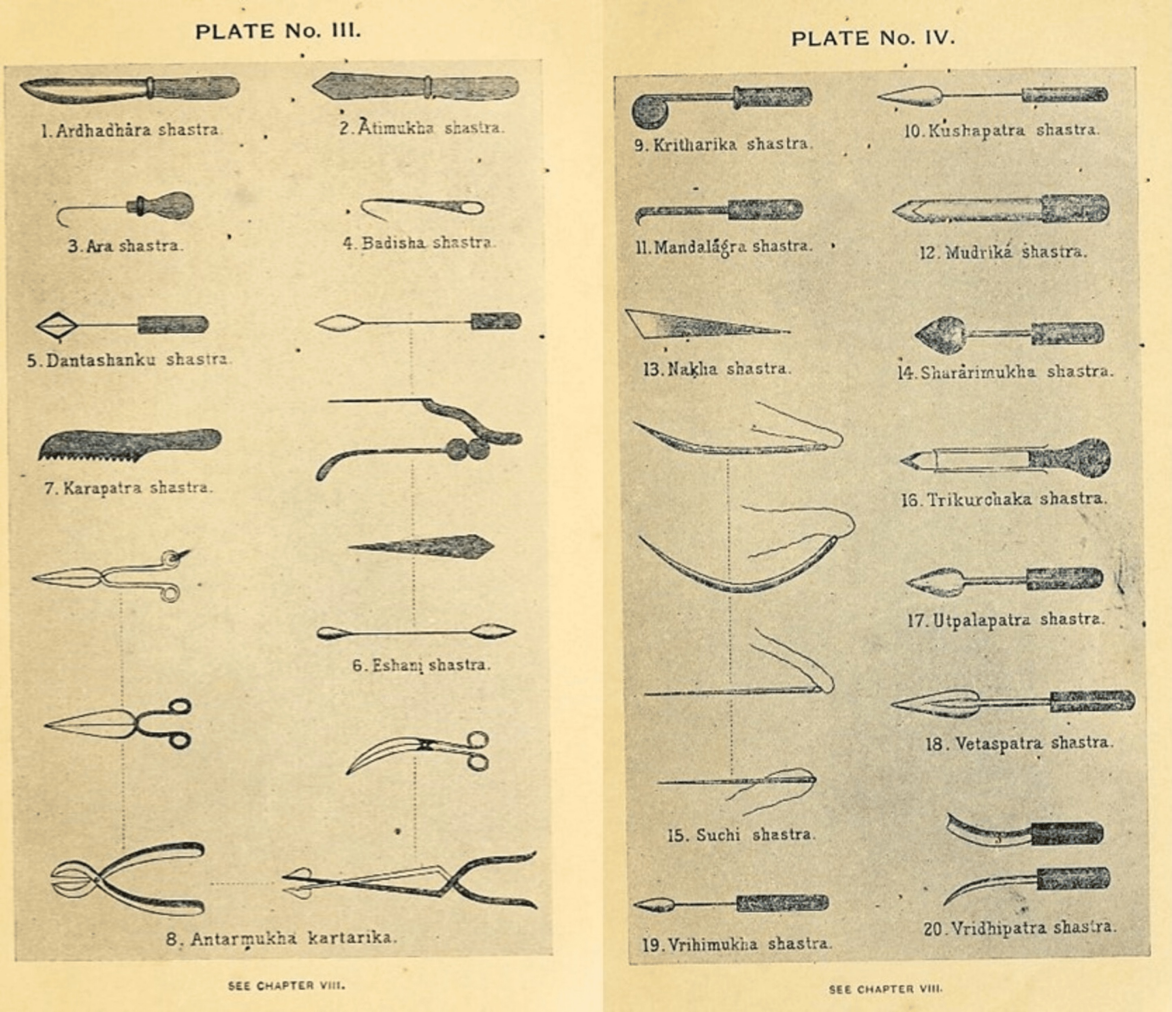
Shastras (Sharp Instruments) invented by Aacharya Sushruta Image source: Screenshots adapted from An English Translation of the Sushruta Samhita, by Kaviraj Kunja Lal Bhishagratna [3], with modifications by the author.

Sushruta's understanding of the human body and surgical precision is evident from the diverse range of instruments he designed. He meticulously labelled and described 121 surgical instruments [10,11], categorized into Yantras (blunt instruments) and Shastras (sharp instruments) (Figures 1-3).

Sushruta's instruments were crafted from iron and other metals available at the time, with an emphasis on cleanliness and sharpness. He recommended regular sharpening and sterilization, aligning with modern-day surgical practices. His designs were characterized with the aid of precision and practicality, with each instrument tailored for specific tasks such as incisions, tissue manipulation, or suturing [11]. Scalpels were designed to make clean cuts, minimizing harm to surrounding tissues, while forceps came in various shapes and sizes for distinct surgical needs. Specialized needles and threads were used for effective wound closure and healing. Probes explored wounds or cavities and hooks retracted tissues to improve visibility during surgical operation. Cauteries were used to govern bleeding and treat conditions by burning or searing tissue, which helped reduce blood loss and seal healing.

Sushruta's innovations in instrument layout included ergonomic considerations to enhance ease of use, and he used a variety of materials, together with metals, wood, and bone, based totally on every device's motive [12]. Some equipment was customized for specific surgeries, reflecting his sophisticated approach. His work had a profound impact on surgical techniques, promoting standardization and improving consequences. Sushruta's instruments and strategies influenced later generations, serving as a model for surgical practices in various cultures and advancing the sphere of medicine.

Surgical techniques (*Shastrakarma*)

Sushruta's surgical techniques were advanced and sundry, covering an extensive range of operations from simple incisions to complex reconstructive surgeries. He categorized his techniques into numerous sorts, ensuring that each procedure was performed with precision and care [12].

Sushruta believed that a successful surgery required not only technical expertise but also an in-depth knowledge of the patient's ordinary health that emphasized the importance of patient care before, during, and after surgery. His strategies were based on the principles of Ayurveda, which emphasized balance and harmony in the body.

Acharya Sushruta's *Shastrakarma *(surgical operations) provides a detailed understanding of ancient surgical procedures, emphasizing both precision and expertise in handling various surgical tools. He classified surgery into eight types of Shastrakarma.

Chedana (Excision)

Sushruta excelled in the *Chedana *(excision) technique, detailing precise incisions with minimal tissue damage, and diseased parts should be removed from the body [13]. His knowledge of anatomy was evident in his use of specialized instruments and attention to incision depth and direction. For this procedure, he used instruments such as *Mandalagra*,* Karpatra*,* Vriddhipatra*,* Nakhasastra*,* Mudrika*,* Utpala Patra*,* *and *Ardhadhara* [11,14].

Bhedana (Incision)

In *Bhedana* procedures, which involved the removal of diseased tissue or foreign bodies, such as pus and *rakta*, by opening or taping the cavity, Sushruta stressed the significance of distinguishing between healthy and unhealthy tissues to ensure effective treatment while preserving as much healthy tissue as possible [13].*Vriddhipatra, Nakhasastra, Mudrika, Utpalapatra, *and* Ardhadhara* were used for *Bhedana*. His teachings prolonged postoperative care, ensuring proper healing and minimizing the risk of infection.

Lekhana (Scraping)

This *Shastrakarma *involves the scraping of superficial tissue, debris, or foreign substances from affected areas in the direction of hair follicles (*Anuloma Gati*) to minimize pain and bleeding while effectively treating conditions such as ulcers, skin diseases, and abscesses [14]. Specialized instruments such as the *Mandalagra *and *Karapatra *were employed for this precise procedure, ensuring careful removal of unwanted material without damaging healthy tissue and preventing further infection.

Vedhana (Puncturing)

*Vedhana* is a surgical procedure that involves making precise punctures in tissues to drain accumulated fluids and toxins from the body, such as in cases of hydrocele, ascites, or other conditions involving fluid buildup. Sushruta emphasized the importance of careful puncturing to avoid excessive damage to surrounding tissues, using specialized instruments such as the *Kutharika, Vrihimukha, Ara, Vetasapatra, *and *Suci* [14].

Eshana (Probing)

*Eshana* is a technique in Sushruta's surgical repertoire used to explore and diagnose wounds or cavities to detect foreign objects, assess damage, or guide further treatment. This procedure is particular for identifying hidden debris, assessing the extent of injury, or locating materials obstructing the healing process and indicating conditions such as* Nadi Vrana* (sinuses), *Sasalya Vrana*, and *Unmargi Vrana* (ulcers with foreign bodies), especially when these wounds follow abnormal directions. The technique involves using the *Eshani *(sharp probe) to carefully explore the affected area [11]. In practice, the probe is held at its base and inserted to navigate through the wound or cavity.

Aharana (Extraction)

This involves the removal of foreign bodies or obstructions such as dental tartar, bladder stones, or accumulated faeces. Techniques include using instruments such as the *Badisa *(sharp hook) and *Dantasamku *(tooth scaler). For bladder stones, the patient is positioned in lithotomy, and the stone is extracted through an incision with specialized tools. Post-procedure care involves a diet of warm rice and medicated preparations to support healing.

Visravana (Draining)

*Visravana *involves the controlled release of accumulated fluids, pus, or other pathological substances to alleviate swelling and promote healing. It is indicated for various conditions, including inflammatory swellings (*Vidradhi*), localized infections (*Ekadeshaja Sopha*), and disorders such as tooth caries and pyorrhea. The instruments used include *Suci, Kushapatra, Atimukha, Sararimukha, Antarmukha, *and *Trikurcaka.* The procedure typically involves creating a small incision or applying leeches to drain the fluid, followed by antiseptic treatment to prevent infection and facilitate recovery. For conditions such as *Vidarika *or *Upadamsha*, leech therapy or direct puncturing might be used depending on the severity and location of the disease.

Seevana (Suturing)

This is a critical surgical procedure aimed at closing wounds with needles and threads to facilitate healing. This procedure is crucial for proper tissue apposition and wound closure. It is indicated for cut wounds, *Sulekhitha Vrana* and localized diseases affecting *Chala Sandhi*. Sushruta emphasized that suturing should be avoided in wounds affected by infection, foreign bodies, or harmful substances. He described different types of suturing needles and techniques, including continuous, interlocking, zigzag, and interrupted methods. The choice of suturing material, such as natural threads or silk, depends on the wound type and location, and careful attention is needed to avoid complications.

Surgical procedures

Sushruta's procedures had given pioneering contributions to surgery and medical practice beyond the basic classification of surgical procedures (Shastrakarma). These encompass a wide range of therapeutic interventions and advanced surgical techniques. Some of them are listed below:

Cauterization (Agnikarma) and Bloodletting (Raktamokshana)

Sushruta's adoption of *Agnikarma *(cauterization) and *Raktamokshana *(bloodletting) proves his well-established understanding of therapeutic interventions. Agnikarma, the application of heat to treat wounds and chronic pain, which sterilizes the wound, prevents bleeding by sealing blood vessels and promotes healing through thermal stimulation, all while minimizing tissue damage [15]. Meanwhile, *Raktamokshana *removes excess blood and balances the bodily humor, treating conditions such as fever, inflammation, and infections. This technique involved methods such as venesection, leeching, or the use of specialized instruments, depending on the condition being treated.

Reconstructive Surgery

**Figure 4:**
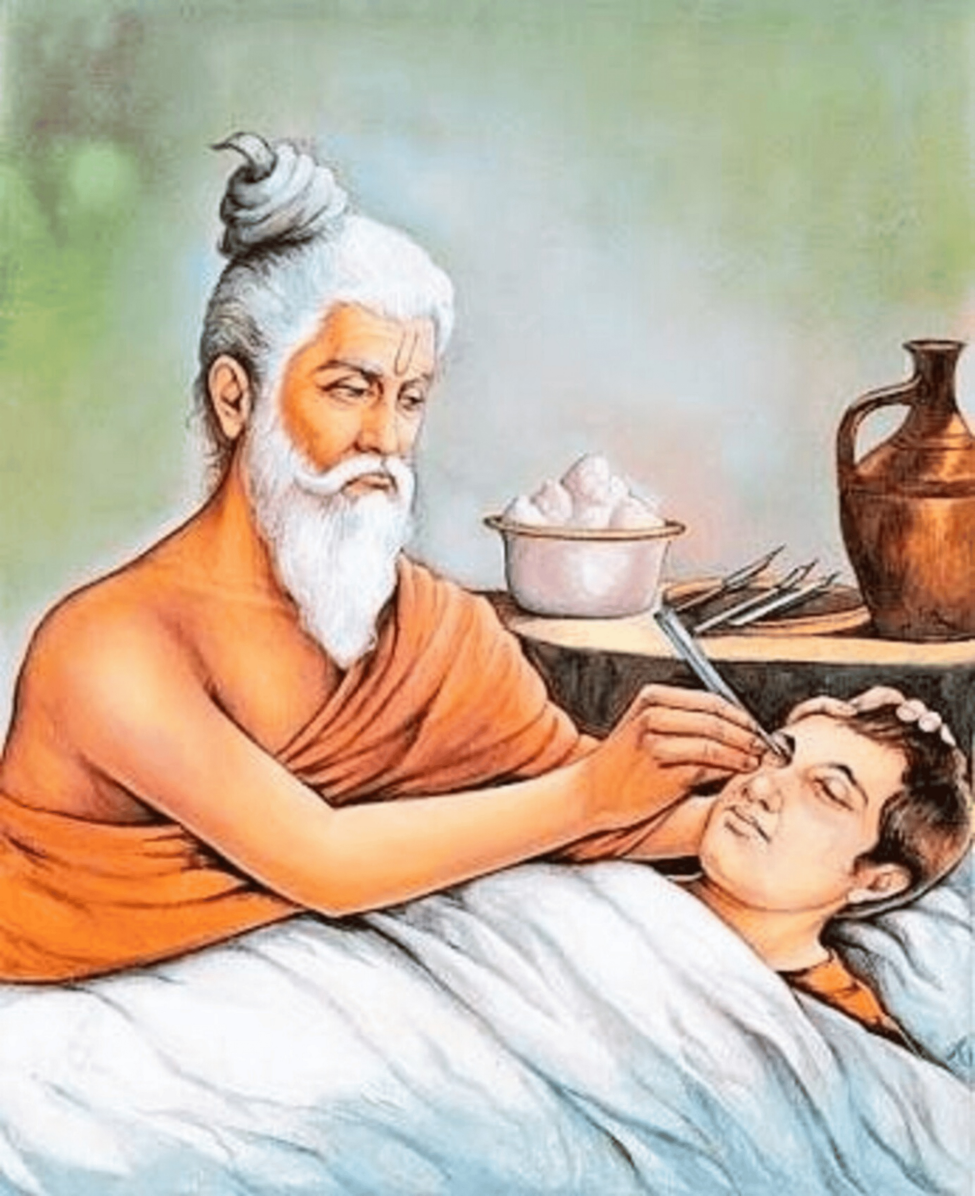
Acharya Sushruta (father of cosmetic surgery) Image source: [17].

Sushruta is renowned for his pioneering work in rhinoplasty (Figure 4), which laid the foundation for modern plastic surgery [16]. In ancient India, nose amputations were a common punishment, leading to severe social and psychological trauma. Sushruta's revolutionary rhinoplasty technique provided a solution by restoring both the physical appearance and dignity of those affected. His procedure, known as the "forehead flap" or "Indian method," involved using a flap of skin from the forehead to reconstruct the nose, emphasizing surgical precision and postoperative care [4]. This technique, still used today in modified forms, attests to his surgical acumen.

Bone Setting and Fracture Management

Sushruta's methods for treating fractures were advanced, classifying them into the following types: simple, compound, and complicated. He emphasized proper bone alignment (*Sandhana*), advocating for reduction (*Sthapana*) through manual manipulation, using splints crafted from bark, bamboo, or cloth, tailored to the fracture's location and severity. His innovative approach to fracture management included post-treatment care, such as a nutrient-rich diet and gentle exercises, laying the groundwork for modern orthopaedic practices [12].

Suturing and Wound Management

Sushruta advocated the use of materials such as horsehair and plant fibres for sutures and emphasized wound cleaning (*Shodhana*) with antiseptic solutions and herbal preparations to prevent infection. This was followed by a meticulous wound assessment (*Roga Pariksha*) to determine the appropriate suturing technique. He described various suturing methods, including interruption sutures (*Vichinna Sutra*) for deep wounds and continuous sutures (*Anuloma Sutra*) for strength and stability, using specialized needles and tools such as forceps and needle holders [18].

He managed complications such as infection, wound dehiscence, and hematomas with re-suturing and herbal treatments when necessary [12]. His approach extended to post-suturing care (*Paricharya*), emphasizing rest, immobilization, regular monitoring, and a nutrient-rich diet to ensure proper healing.

Use of Anaesthesia

Sushruta's approach to anaesthesia, although not as advanced as modern techniques, was innovative. He used herbal anaesthetics such as opium (*Ahiphena*) and cannabis (*Vijaya*) to manage pain, employing methods such as inhalation, oral consumption, and topical application [1,2].

Proper preparation and dosage were crucial, and Sushruta provided detailed guidelines for extracting and administering these herbal remedies [17]. He also employed complementary techniques, such as distraction and positioning, to enhance patient comfort. Despite limitations such as variable efficacy and the short duration of these anaesthetics, Sushruta's methods laid an early foundation for the pain management practices seen in modern surgery.

Cataract Surgery: Early Ophthalmology

Sushruta's cataract surgical treatment marked a vast development in early ophthalmology. His technique, known as *Couching*, involved the use of a specialized instrument called the *Shalaka *[11], a fine rod used to manually dislodge the cloudy lens and push it to the back of the eye [1,18]. This aimed to clear the visual axis and improve vision despite the procedure's risks and limited effectiveness in restoring perfect sight.

Sushruta emphasized precision, recommending that the surgical procedure be done in well-lit conditions, preferably in the morning, with the patient’s head supported for stability. His comprehensive approach, inclusive of meticulous preoperative preparation, sterilization, and postoperative care using medicinal herbs, influenced destiny traits in ophthalmology.

Obstetrics and Gynaecology

Childbirth and women's health: Sushruta's contributions to obstetrics and gynaecology covered techniques for managing childbirth and women's health. He endorsed *Yoni Vasti *(a method for treating gynaecological disorders) and detailed techniques for performing *Kshara Sutra* (a treatment for female reproductive issues), reflecting his comprehensive approach to women's health.

Embryology and genetic diseases: Sushruta's insights into embryology and genetic diseases, while not as detailed as modern science, provided foundational knowledge. In the Sushruta Samhita, he discussed the stages of fetal development and emphasized the importance of maternal health on fetal well-being [8]. He diagnosed the impact of hereditary factors on fitness, noting that genetic conditions could impact both the mother and the offspring. His observations laid the early basis for information on the effect of genetics on health.

Sushruta Samhita: the golden book of surgical innovations and ancient medicine

In addition to the innovations mentioned above, Sushruta made significant contributions to various fields, including neurosurgery, urology, oral and maxillofacial surgery, and trauma surgery. These contributions are meticulously detailed in the *Sushruta Samhita*, which is one of the oldest and foundational texts in the history of medicine and surgery, attributed to the revered Indian surgeon Sushruta [3]. It contains 186 chapters describing 1120 illnesses, 700 medicinal plants, 64 preparations from mineral sources, and 57 preparations based on animal sources [19,20]. It has been a cornerstone of Ayurvedic medicine along with inspiring modern medicine, concentrating on *Shalya Chikitsa* or surgical treatment; indeed, it is considered the oldest available compilation for learning surgical methods. This treatise belongs to the Dhanvantari tradition and comprises, in essence, the principal Samhita with 120 chapters in Purva-tantra, which is subdivided into *Sutrasthana, Nidana, Sarirasthana, Chikitasathanam, and the Kalpastham* [10], while the Uttara Tantra has 66 chapters [6,21].

**Figure 5:**
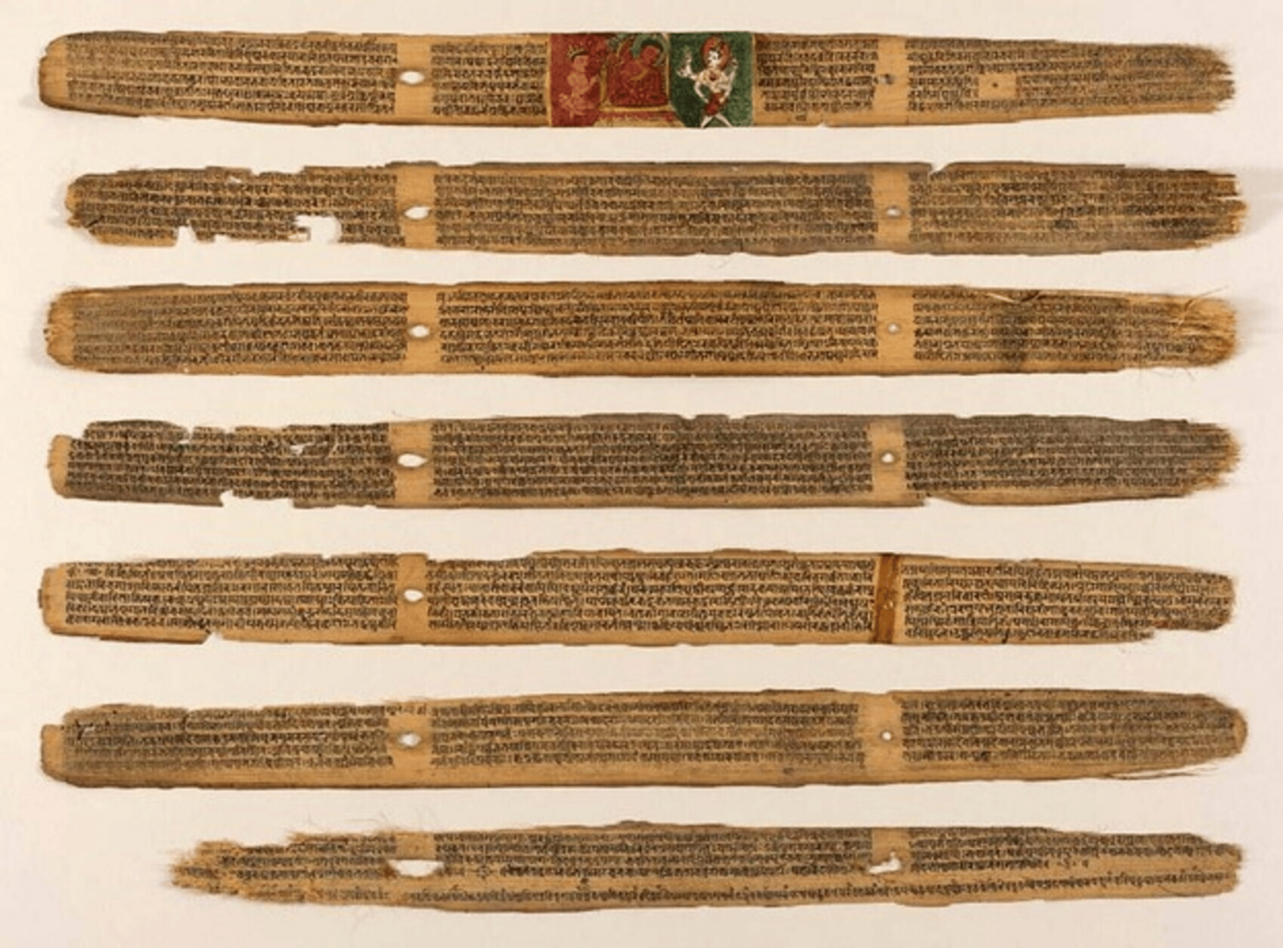
The Sushruta Samhita or Sahottara Tantra (a treatise on Ayurvedic medicine) Image source: public domain

In the Nidana Sthana section, there are 16 chapters dealing with the aetiology, pathogenesis, and clinical features of several diseases, from *Vata *disorders through *Ashmari *(urinary stones) and *Bhagandara *(fistula-in-ano) to rather complex diseases in character [21]. The text further describes several surgical techniques, such as rhinoplasty and cataracts, shedding light on how human anatomy could be so accurately understood through dissection. The scope of the *Sushruta Samhita* extends beyond surgery to questions of general medicine, toxicology, and medicinal plants. Such systematic classification of diseases and their treatments in order of progressing severity testifies to Sushruta's masterly understanding of pathology, making the *Sushruta Samhita* relevant both in historical and contemporary medical contexts (Figure 5).

Influence and legacy

Sushruta is a monumental figure in the history of surgery because of his influence on medicine, which has repercussions transcending time and geography. His pioneering methods not only shaped ancient Indian medicine but also travelled through centuries and continents, impacting medical traditions in Greece, Persia, and Europe [1]. By the 18th century, European surgeons adapted Sushruta's techniques, integrating them into modern plastic surgery.

His principles of precision, cleanliness, and holistic care continue to resonate in contemporary medicine. His rigorous approach to surgical education, combining theoretical knowledge with hands-on practice, laid the basis for modern surgical training. Sushruta’s innovations shaped early concepts of systemic health and continue to inspire and challenge medical practitioners worldwide, reflecting the enduring impact of ancient Indian medical heritage [9].

## Conclusions

Sushruta's remarkable contributions to surgery and medicine laid a foundation that continues to influence modern practice. His unparalleled skill and accuracy in techniques such as rhinoplasty, cataract surgery, and bone setting revolutionized medical practice. Despite the limitations of his era-rudimentary tools and lack of anaesthesia, Sushruta's emphasis on anatomy and comprehensive patient care through autopsy showcased his forward-thinking approach. His principles of precision and cleanliness have endured for centuries, shaping medical traditions worldwide. Sushruta's legacy not only transformed ancient surgery but also established enduring standards in the profession, underscoring his pivotal role in medicine throughout history. Today, his influence continues to inspire and shape both ancient and contemporary medical practices, remaining a cornerstone in the history of surgery.
